# Design and Development of Large-Band Dual-MSFA Sensor Camera for Precision Agriculture

**DOI:** 10.3390/s24010064

**Published:** 2023-12-22

**Authors:** Vahid Mohammadi, Pierre Gouton, Matthieu Rossé, Kossi Kuma Katakpe

**Affiliations:** ImViA Laboratory, UFR Sciences et Techniques, University of Burgundy, 21078 Dijon, France; v.mohamadi1@gmail.com (V.M.); matthieu.rosse@u-bourgogne.fr (M.R.); kossi.katakpe@imsp-uac.org (K.K.K.)

**Keywords:** multispectral filter array, multispectral camera, snap-shot spectral camera, color shade technology, demosaicking

## Abstract

The optimal design and construction of multispectral cameras can remarkably reduce the costs of spectral imaging systems and efficiently decrease the amount of image processing and analysis required. Also, multispectral imaging provides effective imaging information through higher-resolution images. This study aimed to develop novel, multispectral cameras based on Fabry–Pérot technology for agricultural applications such as plant/weed separation, ripeness estimation, and disease detection. Two multispectral cameras were developed, covering visible and near-infrared ranges from 380 nm to 950 nm. A monochrome image sensor with a resolution of 1600 × 1200 pixels was used, and two multispectral filter arrays were developed and mounted on the sensors. The filter pitch was 4.5 μm, and each multispectral filter array consisted of eight bands. Band selection was performed using a genetic algorithm. For VIS and NIR filters, maximum RMS values of 0.0740 and 0.0986 were obtained, respectively. The spectral response of the filters in VIS was significant; however, in NIR, the spectral response of the filters after 830 nm decreased by half. In total, these cameras provided 16 spectral images in high resolution for agricultural purposes.

## 1. Introduction

Single-shot (also called snap-shot) multi-spectral (MS) cameras contain multi-spectral filter arrays (MSFAs) composed of several spectral filters (normally more than five filters). These filters are narrow-band filters that provide extra information compared to common RGB cameras [1]. MS cameras provide more information, as they provide images in different regions of the electromagnetic spectrum [2]. The classical solution was to use a filter wheel in front of an image sensor. Each time that a bandpass filter was in front of the image sensor, an image was captured. Another solution was to use passband filters on different image sensors and different optics. However, the recent solution proposes that MSFAs be used on the top of the image sensor to provide a MS image in a single-shot [3].

Previous research has shown that, for agricultural applications, most of the necessary information is obtained in the visible (VIS) and near-infrared (NIR) parts of the spectrum [4,5,6,7,8,9]. In this regard, much research has been conducted on the use of VIS and NIR spectral data, such as the development of spectral indices [10,11,12], the detection of plant diseases [13,14,15,16], weed detection [7,17,18], nutrient content estimation [19,20], monitoring water stress [21], phenotyping [22,23], and the quality measurement of agricultural products [24,25]. Thomas et al. [26], in their study on the benefits of hyperspectral imaging for disease detection, pointed out that, in addition to the visible part of spectral data (i.e., 400–700 nm), the NIR wavelengths (i.e., 700–1000 nm) significantly represent the pathogens influencing the cellular structure of plants. Shapira et al. [27] used spectroscopy for weed detection in wheat and chickpea fields in the range of 400 to 2400 nm. They reported 11 wavelengths as the most effective wavelengths for weed detection, all of which fell into the range of 400–1070 nm.

MS imaging brings about many advantages compared to other spectral techniques. Spectroradiometers have been used in previous research; however, spectroradiometers are highly expensive and sensitive tools that provide data only for a single point. Another difficulty is dimensionality reduction, as the spectroradiometers provide a huge amount of data. But MS imaging provides the data via effective spectral bands, which directly helps the extraction of useful features for analysis. In addition, the MS imaging provides the spectral data for a region in a snap-shot that is necessary for a wide range of applications. Thus, novel snap-shot MS cameras can replace the usage of spectroradiometers, facilitate agricultural production and operation management, reduce costs of spectral measurements, and decrease the amount of hyperspectral data analysis.

Recently, much attention has been paid to MS imaging, and several works have been reported for the development of MSFA, MS cameras, and demosaicking algorithms [28,29,30,31,32]. Abbas et al. [33] reported a novel technique for the demosaicking of spectral snap-shot images. They reported a better performance compared to the previous technique. However, recent research on demosaicking techniques has been focused on the use of deep learning applications [34,35,36]. Ma et al. [37] proposed a near-infrared hyperspectral demosaicking method which was based on convolutional neural networks. The method was developed for low illumination and a mosaic of 5 × 5 pixels.

Research on the development of MSFA cameras has been oriented toward low-cost, compact, snap-shot, and high-resolution cameras. However, improving the resolution, increasing the number of bands, and providing snap-shot imaging remarkably increases the costs of these cameras. Sun et al. [38] developed a four-band MS camera based on MSFA technology. They used an array of 2 × 2 and proposed a demosaicking algorithm based on directional filtering and wavelet transform. As another solution, Bolton et al. [30] developed a MS imaging system for VIS and NIR based on the use of a monochrome camera and 13 different LEDs with different working areas in VIS and NIR. Cao et al. [39] developed an artificial compound eye (ACE) for MS imaging. The center wavelengths were 445 nm, 532 nm, and 650 nm, and the imaging systems were made up of combinations of diffractive beam splitting lenses. They concluded that, based on the structure of the system, which was multi-aperture and composed of multiple sub-eye lenses, different kinds of spectral imaging structures with various central wavelengths would be possible to design. He et al. [40] developed a three-band MS camera using a filter array integrated on the monochrome CMOS image sensor. Their camera was based on a narrow spectral band, a red–green–blue color mosaic in a Bayer pattern. The proposed MSFA consisted of a hybrid combination of plasmonic color filters and a heterostructured dielectric. 

In this work, two MS cameras covering VIS and NIR ranges were designed and developed for agricultural applications. The spectral resolution of these cameras was several times higher than that in previous research, as 16 spectral bands were provided for VIS and NIR. The optimized spectral bands were selected using a genetic algorithm between 380 nm and 950 nm based on the spectral reflectance of eight plants and weeds. A novel design for filter arrays was proposed for the MS cameras, with eight spectral bands in a mosaic of 4 × 4 pixels. The MSFAs were developed based on Fabry–Pérot technology. A commercial CMOS sensor was chosen, and specific sensor boards were developed.

## 2. Materials and Methods

The development of novel MS cameras requires the creation of MSFAs, the design and development of a sensor board, the assembly of the MSFA, and the development image sensor and software. Hereafter, these steps will be explained in different sections.

### 2.1. Image Sensor

The CMOS sensor is the physical element whose performance impacts the quality of the final imaging system. In this project, the chosen sensor needed to meet the criteria below in order to achieve the best performance for this application:

- Minimum pixel size needed to be greater than 4 µm; this was a limitation caused by the COLOR SHADES^®^ Filter technology, which was used to create the MSFA filters;
- The CMOS sensor resolution had to be high enough to compensate the loss related to the MSFA system (i.e., sensor resolution > 1 k);
- The spectral sensitivity of the sensor was required to be extended to the near-infrared range (i.e., 380–1000 nm);
- Quantum efficiency was very good in the chosen spectral band;
- Integration time was variable.

Taking into account the above specifications, our choice was the E2V Sapphire sensor which is a grey-scale CMOS sensor without cover glass. This sensor was provided by E2V Teledyne Company, Saint-Egrève, France. Table 1 provides the technical details of the chosen image sensor.

### 2.2. Band Selection

The goal of this project was to provide a sensor based on MSFA technology that covered a spectral range between 380 and 1000 nm. In this regard, an MSFA was needed for the visible range and an MSFA for the NIR. Thus, the spectral area of interest could be divided into two zones:

- The area was centered around 550 nm and was all-around green, the most important color in the field;
- The area of near infrared, which was promising for the estimations based on the amount of chlorophyll in the plant leaves.

For band selection, a dataset of 626 spectral reflectance samples of eight plants, including three crop plants (i.e., tomato, cucumber, and bell pepper) and five weeds (i.e., bindweed, nutsedge, Plantago lanceolata, potentilla, and sorrel) was prepared and used via the band selection program. The spectral reflectance was measured using the spectroradiometer Specbos 1211 (JETI Technische Instrumente GmbH, Jena, Germany) under a type A illuminant. 

The optimized selection of spectral bands is a difficult task, and thousands of combinations are possible. Accordingly, the band selection was conducted using Genetic Algorithm (GA). The program was prepared using MATLAB software (2019b version, MathWorks Inc., Natick, Massachusetts, USA). Considering *k* samples and *i* (*i* = 1, 2, …, *n_u_*) multispectral filters, the response of a CMOS sensor, *O*, could be expressed as follows:

(1) $$O_{i,k}={SU}_{i}{Er}_{k}$$

where *S* is the sensitivity of the CMOS sensor, *U_i_* is the diagonal matrix of spectral transmittance for the *i*th filter, *E* is the diagonal matrix of spectral power distribution of an illuminant, and $r_{k}$ is the spectral reflectance of the *k*th sample in the data set. The reconstruction of spectral reflectance $r_{k}$ was achieved via Equation (2):

(2) $$r_{k}=WO_{i,k}$$

where *W* is the Wiener transformation matrix. The Gaussian model, which is a continuous and symmetric distribution, was used as follows for the simulations of the transmission of interference filters [41]:

(3) $$u_{\lambda }=\alpha .\frac{1}{\sigma \sqrt{2\pi }}\;.\;e^{-\frac{1}{2}(\frac{\lambda k-\mu^{2}}{\sigma })}$$

where μ and σ control the center and width of the filter, λ stands for the wavelength, and α and *k* are constants. The SNR was chosen as 100, adding normally distributed white noise to the system. FWHM (filter width at half maximum) was set to 30 nm for both VIS and NIR. The fitness limit, crossover fraction, and migration fraction were set at 0.02, 0.6, and 0.2, respectively. The population size was determined to be 100.

### 2.3. MSFA Sensor Technology

**Figure 1:**
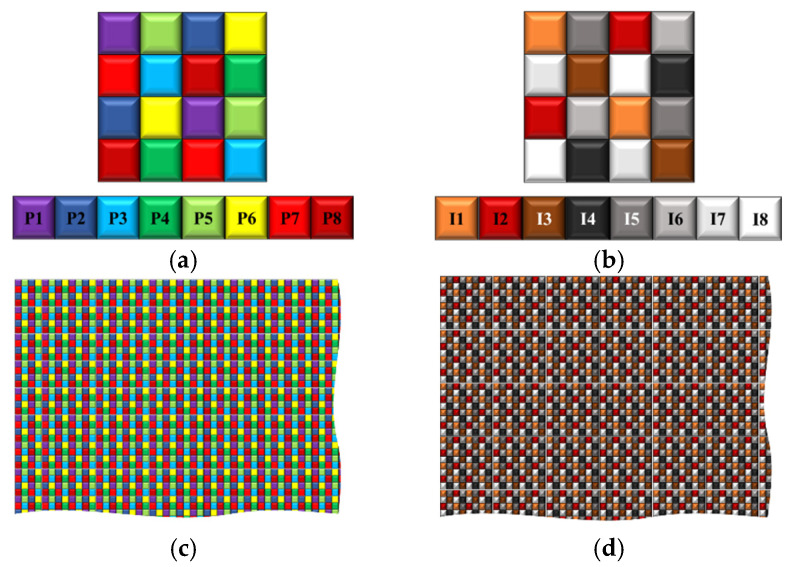
Distribution of the final moxel. (**a**) VIS, (**b**) NIR, (**c**) cross-section of VIS MSFA, and (**d**) cross-section of NIR MSFA.

The development of MSFA was carried out using color shade technology. In this work, the VIS camera was equipped with an MSFA of 8 bands, covering from 380 to 680 nm (Figure 1). The NIR camera was equipped with an MSFA of 8 bands, started from 650 to 900 nm (Figure 1). This solution is often used with several cameras, where each camera is equipped with one multi-band filter array. Despite the ease of implementation of this solution, it presented some challenges, one of which was that each camera had its own optical axis. This poses a problem in the reconstruction of multi-band images. Also, it is difficult to properly manage the synchronization of the cameras. One important advantage is the ability to manage the integration time of the two cameras separately based on their sensitivity. 

Based on our experiences in MSFA imaging, some modifications were introduced in order to improve the final sensor [42]. The modification was a change that was made at the level of the moxel (i.e., filter pixel array). Indeed, to compensate for the loss of sensitivity beyond 850 nm, a complex structure of the moxel (4 × 4 pixels) was utilized (Figure 1).

### 2.4. Hybridation of MSFA and Image Sensor

**Figure 2:**
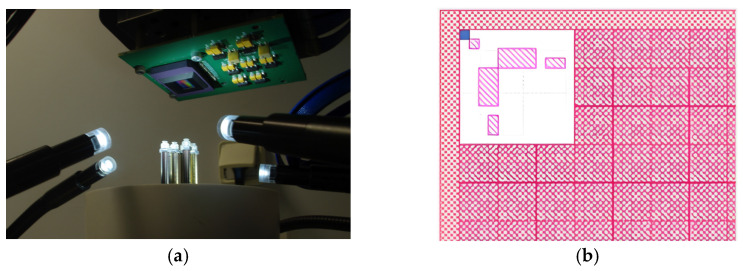
Sensor hybridation: (**a**) the central part of the nano-positioning system and (**b**) alignment marks on the top left corner of the filter plane.

**Figure 3:**
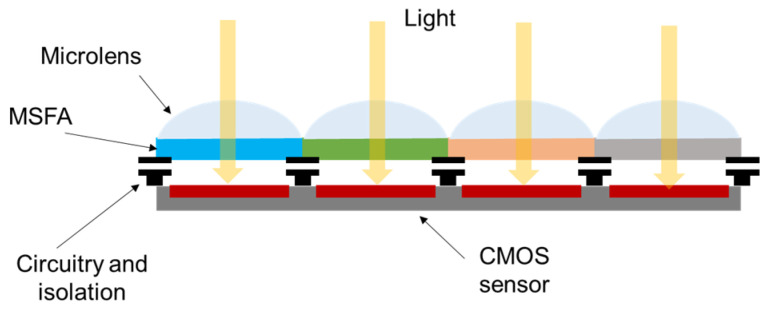
Structure of the hybrid sensor.

Hybridation is the assembly process of the MSFA on the CMOS sensor. This assembly brings about difficulties which have been observed in previous projects [43]. The most important problem is crosstalk, which stands for the misplacement of filter units on the image sensor pixels. This mismatch between a pixel and its correspondent filter unit leads to the passing of unfavorable light or partial blocking of light. For this hybridation, a nano-positioning system (6D ALIO, Arvada, CO, USA) was used. Figure 2a shows the central part of this system, in which the CMOS sensor is attached to the top part and the MSFA is held to the system by the supports. Using the alignment marks placed in the 4 corners of the MSFA plane, the CMOS was displaced and positioned on the MSFA plane (Figure 2b). The final sensor was a snap-shot MFSA camera sensor (Figure 3).

The sensor, integrated into a camera with dedicated hardware and software, allowed for the real-time operation of applications at 30 fps. The increase in the camera speed had an impact on the spatial resolution. In order to provide an optimal solution for the loss of spatial resolution inherent to the MSFA, specific algorithms for multispectral demosaicking were developed.

### 2.5. Driving Board

This unit was responsible for receiving data from the sensor board, the control unit, the processing data, and the communication unit with a PC or other devices. The design was carried out using the Vivado platform (Vivado version 2018.2) of Xilinx, which is a graphical development environment. The algorithm representing the functionality of this unit was as follows:

- Receive the video stream (48-bit) from the sensor board;
- De-serialize the data into a single stream;
- Detect the pixel flow (timing detector);
- Control and prepare the data for simultaneous input and output;
- Generate a pixel flow (timing generator);
- Send data to communication gates (HDMI/VGA).

### 2.6. Sensor Analysis and Characterization

To estimate the spectral response of the filter, a global estimation of the filter was carried out. This was achieved using a monochromator system (Gooch and Housego, Orlando, FL, USA) present in the PImRob Platform of the ImViA. This system has a full-width half-maximum (FWHM) of 0.25 nm. The wavelengths of the incoming light were swept in steps of 5 nm from 300 nm to 1000 nm. An image was captured for each wavelength with a proper integration time, which permitted no saturation of any of the channels, but maximized the incoming signal to limit noise. Some information was required to be verified during this calibration process, as follows:

- Verifying energy balancing, which is important in order to provide the same factors for a polychromatic light so that a software correction can be provided;
- Analyzing the impact of rejecting bands, which sometimes occur when using the Fabry–Pérot system. In this case, the impact of the addition of a band pass filter to the hybrid sensor needed to be analyzed;
- Analysis of the MSFA positioning impact and the crosstalk between the MSFA and the image sensor;
- Considering the need to amplify the signal or change the nature of the substrate used for MSFA development.

Energy balance is important for single-sensor spectral imaging in order to minimize the noise and balance it between channels. Indeed, it might happen that one channel is saturated while another does not receive enough incoming light, which would critically impair the application [42]. We call $\rho_{p}$ the response of the camera according to a simple model of image formation, assuming the perfect diffuser reflectance, as defined in Equation (4) [42]:

(4) $$\rho_{p}=\int_{\lambda min}^{\lambda max}I\left(\lambda \right)S_{p}d\lambda$$

where *I(λ)* is the spectral emission of the tested illuminant, *S(λ)* is the camera response, and *p* is the index of the spectral band. The standard illuminants of the CIE (Commission Internationale de l’Eclairage) could not be used for the NIR part of the spectrum, as they could not yet be described by these standards. Thus, alternative illuminations were selected and computed. Therefore, a D65 solar emission simulator was selected. In addition, tungsten and illuminate E were considered. To estimate the spectral response of the filter, a global estimation of the MS imaging sensor was set up. To realize this calibration, the following steps were defined. First, the energy generated by the monochromator was measured from 360 to 800 nm for the VIS Sensor and from 550 to 1050 nm for the NIR. Second, the system generated a monochromatic light each 5 nm from 360 to 1100 nm. Thus, the original CMOS sensor (without a mounted filter) was illuminated. The integrated time of the camera was set as constant. In this way, the spectral response of the sensor was measured. The second measurement was made with the MS imaging sensor. An image processing algorithm was built to extract a pixel related to a specific filter from each moxel. Finally, the filter’s spectral response was obtained from the CMOS sensor response measured in the second step.

### 2.7. Assembly and Build-Up

**Figure 4:**
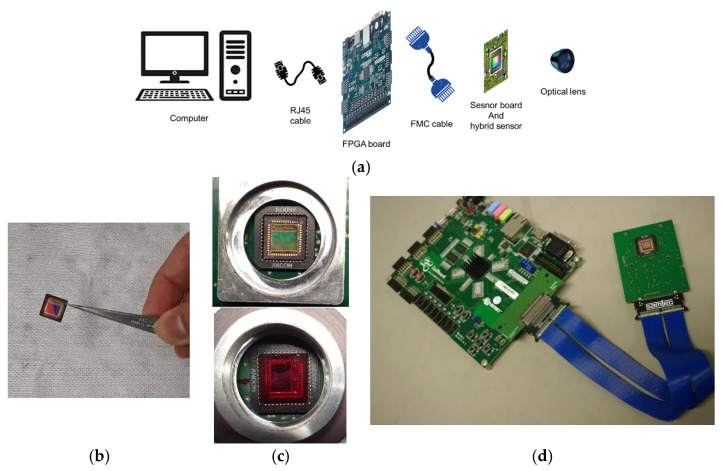
The overall structure and different components of the system. (**a**) All of the components before assembly, (**b**) the CMOS sensor, (**c**) CMOS with MSFA mounted on the board (the VIS sensor above and the NIR sensor below), and (**d**) the assembled components and boards.

Figure 4 represents the components of the MSFA system. There are three main parts, including the MSFA sensor and its board, the FPGA board, and the windows-based application. The developed sensor board was connected to a Zedboard (Digilent FPGA) via a deported and very flexible connection. For the connection, an FMC cable with 100 pins was used. This unit was designed to control signals coming from the sensor, so a link between the processor part and the PL part was necessary. The Zedboard was connected to the PC using an RJ45 cable.

### 2.8. Demosaicking and Image Reconstruction

**Figure 5:**
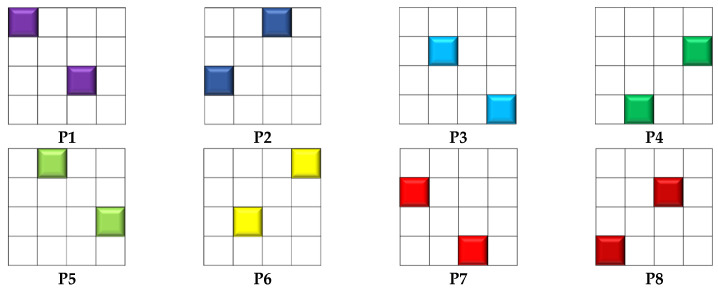
Mosaic masks and the pixels whose values need to be determined.

Each monochrome image taken by the cameras consisted of data on 8 single-band planes. The demosaicking procedure refers to the extraction and reconstruction of an MS image consisting of eight single-band images (i.e., an image cube of eight layers). The demosaicking algorithm read the monochrome image, took each mosaic of the image, completed the missing pixels, and produced an image of the same size. Figure 5 illustrates the architecture of a single mosaic and how two pixels were attributed to each filter. These masks were repeated *N_l_* and *N_c_* times along lines and columns of the image to cover the entire image.

*N_l_* = 1200/4 = 300

*N_c_* = 1600/4 = 400

Bilinear demosaicking was applied on each band. This included finding the missing values using linear interpolation in both main directions. The final step was to calibrate the reconstructed image based on the standard Lambertian white.

## 3. Results and Discussion

### 3.1. CMOS Sensitivity

**Figure 6:**
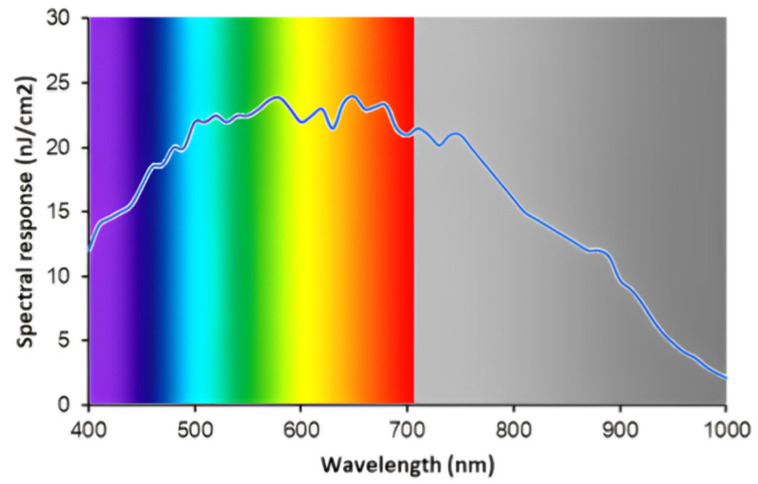
Spectral response of E2V image sensor.

The sensitivity of the CMOS sensor was evaluated. Figure 6 shows the spectral response of this sensor, which should be taken into consideration for band selection and filter development. The sensor was provided by Teledyne E2V (EV76C560, Chelmsford, UK). Although the gain fell toward the IR, which makes sense for an SI sensor, it was still acceptable. 

### 3.2. Spectral Bands

The spectral bands for the creation of MSFAs were determined using a simulation program based on GA. Table 2 presents the details of the simulations for band selection in VIS and NIR, obtaining goodness of fitness coefficients (GFCs) of 99% and 98%, respectively, at the probability level of 99%. The standard deviation was quite small, and the achieved RMS values were 0.012 and 0.011 for VIS and NIR, respectively. Hence, using a genetic algorithm, the sixteen optimized Gaussian filters were obtained based on the fitness function of Wiener.

**Figure 7:**
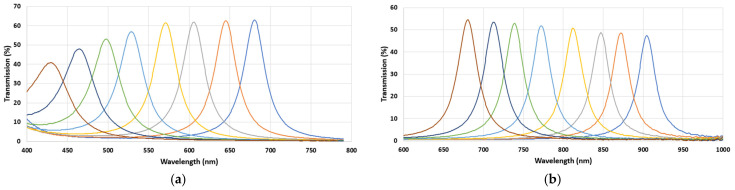
Spectral sensitivities of the MSFAs measured before the hybridation on the sensor; (**a**) VIS and (**b**) NIR.

Figure 7 illustrates the spectral sensitivities of the chosen filters after fabrication. This figure presents the relative sensitivities of each filter. It can be seen that the transmission rates of all bands were over 40%, and very good consistency was observed for the primary prototypes.

The bands selected through the simulation and the final, real results are shown in Table 3 and Table 4. It is observed that the actual bands after fabrication were slightly different from the desired bands; however, this is normal as a result of the production process of the filters. The choice of bands plays a critical role in the development of MS cameras, as it directly depends on the application of the MS cameras and their functionalities. The final spectral bands of the MSFAs perfectly match agricultural applications such as disease detection. Yang et al. [44] reported 426 nm as the effective wavelength in the spectral detection of brown plant hopper and leaf folder infestations of rice canopy. Zhang et al. [45] reviewed the previous research on the remote sensing techniques for disease detection and reported 655 nm and 735 nm as important fluorescence indicators. Huang et al. [46] studied the detection of rice leaf folder based on hyperspectral data. Important disease detection regions were observed at 526–545 nm, 550–568 nm, 581–606 nm, 688–699 nm, 703–715 nm, and 722–770 nm. Khaled et al. [47] studied the early detection of diseases in plant tissue using spectroscopy. They found that the wavelengths of 550 to 630 nm aided in the detection of the disease. The study of Qin et al. [48] on the detection of citrus canker showed that the wavelengths of 553, 677, 718, and 858 nm were the most effective. The usage of MS cameras with a few bands on the effective wavelengths also has been taken into consideration. Stemmler [49] developed an MS camera using an RGB and two monochrome cameras taking MS images in three bands (i.e., 548, 725, and 850 nm). For the monochrome cameras, they took advantage of band-pass interference filters. Du et al. [50] developed a MS imaging system covering a wider range of electromagnetic spectrum. They designed and constructed a common-aperture MS imaging system which took VIS, short-wave infrared (SIR), and mid-wave infrared (MIR) MS images. Their structure was based on two dichroic mirrors for the purpose of splitting the light spectrum.

These tables provide the spectral specifications of the MSFAs. We observed that, except for the filters of 425 and 460 nm, all other filters met the desired FWHM of the specification. For both VIS and NIR MSFAs, the maximum transmission was over 40% which is desired for MS applications. As we observed, the spectral response of the filters was very good, and the best response was achieved in the red region of the spectrum for the VIS and between 650 nm and 750 nm for the NIR.

**Figure 8:**
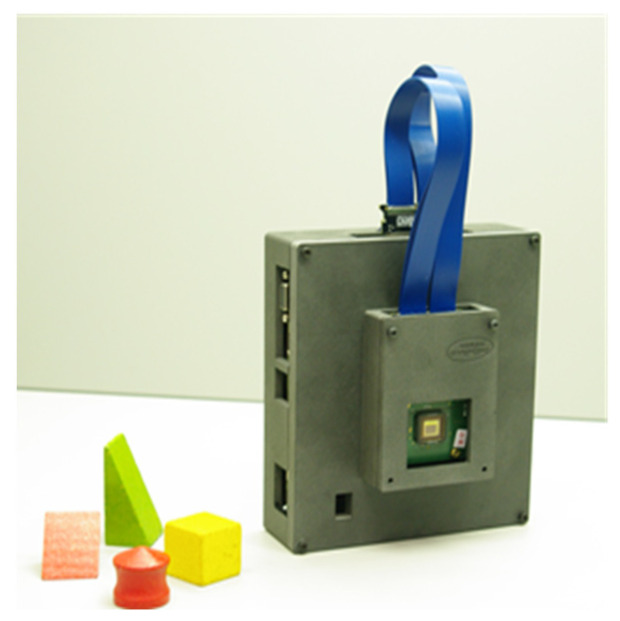
The final camera after assembly of all parts.

After mounting the MSFA on the image sensor, other pieces, including the sensor board, the driving board, the data transfer cables, and the box, were assembled. Figure 8 shows the developed camera. The box, designed via 3D printing, included two main parts: the small part in front for the sensor board and the back part for holding the driving board and other connections.

**Figure 9:**
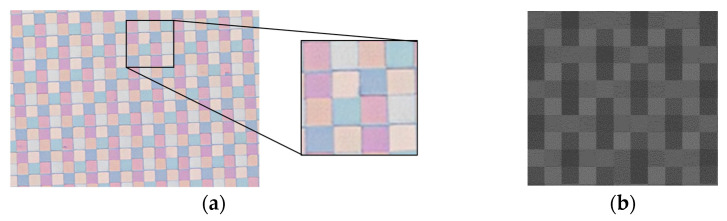
The sensor imaged under a microscope; (**a**) hybrid sensor; (**b**) mosaic image.

**Figure 10:**
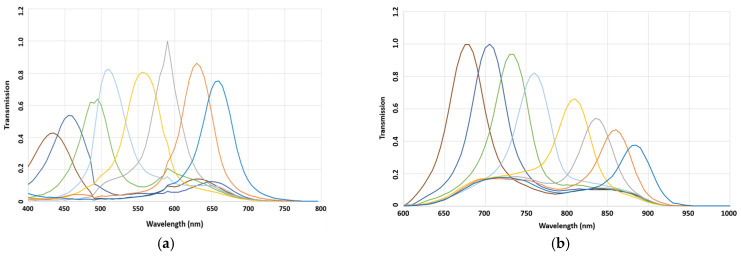
Spectral response of the cameras after hybridation process. (**a**) VIS hybrid sensor; (**b**) NIR hybrid sensor.

A photo of the mounted hybrid sensor under microscope is presented in Figure 9. A mosaic of the MSFA is magnified for verification of the structure and pixels (Figure 9a). Figure 9b presents a zoom-in of image acquired with the one-shot VIS MS camera. Figure 10 illustrates the spectral sensitivity of the MS image sensor. The lowest filter responses were found at 425 nm and 885 nm for the VIS and NIR MSFAs, respectively. 

**Figure 11:**
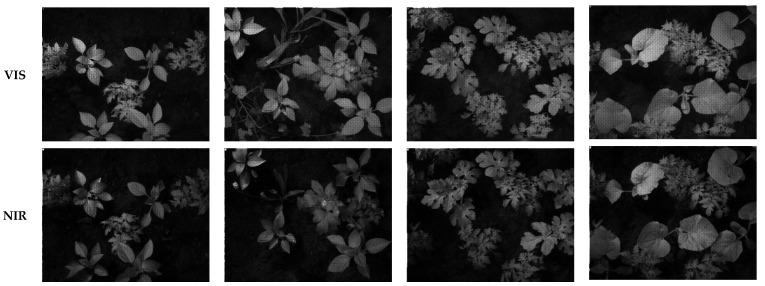
Example images taken by the cameras from different plants. The cameras were mounted beside each other, and the images of the scene were taken by the cameras simultaneously. Top row: VIS image; bottom row: NIR image.

Figure 11 presents some of the images taken by the cameras from several plants. The VIS and NIR images were taken at the same time for the same scenes. These images are the raw images, which include eight single-band images to be extracted by demosaicking. The illumination used for these images consisted of three halogen lights. This illumination was chosen to provide enough energy for both VIS and NIR. Shrestha et al. [51] used a 3D camera with two sensors for an MS camera by using MS filters in front of the image sensors. In a recent study, Zhang et al. [52] developed an MS camera for the visible range. The camera was designed to be compact, handheld, and of high spatial resolution.

**Figure 12:**
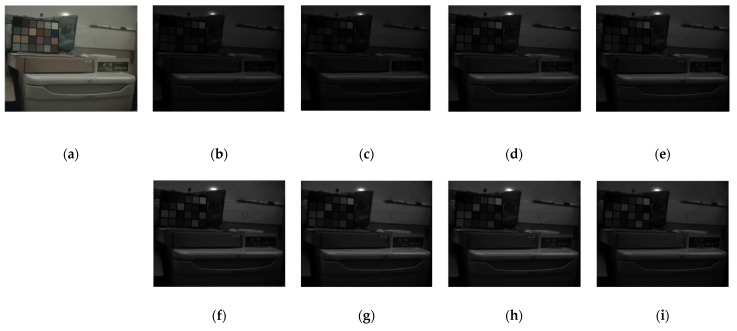
A sample image of a scene with a color checker and the result after demosaicking with the bilinear algorithm. (**a**) The RGB built using three bands, (**b**–**i**) different MS image bands from P1 to P8 channels.

Figure 12 provides an image of a scene with a standard color checker. In this figure, (a) represents a color image built using one of the multispectral cameras, and the rest are eight bands of the VIS MS camera. In this figure, (b)–(i) are images that have been reconstructed by the demosaicking algorithm.

## 4. Conclusions

In this project, two MS cameras were developed for agricultural applications. Each MS camera consisted of an eight-band MSFA, which covered from 380 nm to 950 nm (i.e., VIS and NIR). This type of MS camera, which has relatively good resolution, brings about advantages for many applications in agriculture. 

A novel mosaic of 4×4 pixels was proposed, and it was observed that the proposed mosaic pattern may be promising for snap-shot MS cameras. The pixel pitch decreased in comparison to previous research, leading to a significant outcome. The proposed CMOS sensor provided good spectral sensitivity in VIS and NIR. Spectral band selection was optimized using GA, and a total of 16 spectral bands were selected and proposed for agricultural applications. It was observed that the fabrication process shifted the centering of the filters, but the sensitivity levels of all filters were acceptable. The MSFA was mounted on the image sensor with a nano-positioning system to avoid the crosstalk problem. It was observed that the proposed mosaic pattern and the demosaicking algorithm functioned successfully. The spectral response of the cameras was remarkable, and for both cameras, the MS images were clear, precise, and of a high resolution.

## Figures and Tables

**Table 1:** Electro-optical specifications of the E2V image sensor.

Parameter	Description	Unit	Typical Value
Sensor characteristics	Resolution	pixels	1600 (H) × 1200 (V)
	Image size	mm	7.2 (H) × 5.4 (V)
	Pixel size	μm ^2^	4.5 × 4.5
	Aspect ratio	-	4/3
	Max. frame rate	fps	50
	Pixel rate	Mpixels/s	114 –>120
	Bit depth	bits	10
Timing modes	Global shutter in serial and overlap modes; Rolling shutter and Global Reset modes; Output format 8 or 10 bits parallel plus synchronization; SPI controls; Control input pins: Trigger, Reset; Light control output.		

**Table 2:** Simulation results of band selection using GA.

Parameter	VIS	NIR
Min Delta E 2000	0.0047	0.0051
Max Delta E 2000	0.3500	2.2575
Mean Delta E 2000	0.0562	0.0937
Median E 2000	0.0465	0.0658
STD Delta E 2000	0.0402	0.1320
Min RMS	0.0020	0.0021
Max RMS	0.0740	0.0986
Mean RMS	0.0123	0.0108
GFC > 0.99	99%	98%
GFC > 0.95	100%	100%

**Table 3:** Comparison between the specification and the actual centered wavelengths for the VIS MSFA.

Band	Specification *	Measure **			STD		
	Centering (nm)	Centering (nm)	FWHM (nm)	Tmax (%)	Centering (nm)	FWHM (nm)	Tmax (%)
P1	421	425	71	41	4	36	1
P2	457	460	52	48	3	17	8
P3	493	500	44	53	7	9	13
P4	529	510	39	57	−19	4	17
P5	565	560	36	62	−5	1	22
P6	601	590	34	62	−11	−1	22
P7	637	630	33	63	−7	−2	23
P8	673	660	33	63	−13	−3	23
Average			43	56		−8	16

* Specification centering ± 10 nm (best effort ± 8 nm). ** Specification for FWHM = 35 nm ± 10 nm; Specification for Tmax > 40%.

**Table 4:** Comparison between simulated and real centered wavelengths for the NIR MSFA.

Band	Specification *	Measure **			STD		
	Centering (nm)	Centering (nm)	FWHM (nm)	Tmax (%)	Centering (nm)	FWHM (nm)	Tmax (%)
P1	688	670	31	55	−18	1	15
P2	718	705	30	53	−13	0	13
P3	748	735	30	53	−13	−1	13
P4	778	760	29	52	−18	−2	12
P5	808	810	30	51	2	−1	11
P6	838	840	28	49	2	−3	9
P7	868	860	27	49	−8	−4	9
P8	898	885	27	47	−13	−4	7
Average			29	51		1	11

* Specification centering ± 10 nm (best effort ± 8 nm). ** Specification for FWHM = 35 nm ± 10 nm; Specification for Tmax > 40%.

## Data Availability

Data are contained within the article.
